# The impact of atmospheric pollutants on the physical health of college students based on physical examination data of college students from a university in Xi’an, Shaanxi Province, China

**DOI:** 10.3389/fpubh.2025.1428820

**Published:** 2025-03-24

**Authors:** Jiaxin He, Ke Liu, Zhiyu He

**Affiliations:** ^1^International Business School, Shaanxi Normal University, Xi’an, Shaanxi, China; ^2^Physical Education and Sports Science Center, Xi’an Jiaotong University, Xi’an, Shaanxi, China

**Keywords:** air quality, physical health of college students, health effects, physical measurement data, pulmonary function of college students

## Abstract

**Background:**

Air pollution, particularly particulate matter (PM_2.5_/PM_10_), poses a significant environmental health threat in urban China. While previous research has primarily focused on older adult populations, the impact of air pollution on college students—an important yet underexplored demographic—remains largely unclear. This study investigates the effects of air pollutants on physical fitness and lung function among students at a university in Xi’an, a city known for its persistent air quality challenges.

**Methods:**

We used longitudinal physical examination data (2019–2022) from 21,580 college students to perform empirical correlation regression and kernel density estimation. Trends in physical fitness and vital capacity scores were analyzed alongside air quality indicators (AQI, PM_2.5_, PM_10_, CO). A mixed cross-sectional econometric model controlled for individual characteristics such as height (mean = 170.66 cm, SD = 8.37), weight (mean = 64.94 kg, SD = 13.40), gender (mean = 0.313, SD = 0.464), and environmental factors such as temperature, wind speed, and green coverage (mean = 41.22, SD = 1.45). Physical fitness scores exhibited high variability (SD = 9.62, range = 10.2–109).

**Results:**

Air pollution was significantly associated with a reduction in physical fitness scores. A 1-unit increase in the AQI was linked to a 0.1094-unit decline in fitness scores (*p* < 0.01). The negative effect was further amplified by PM_2.5_ (*β* = −0.2643) and CO (β = −11.5438). Senior students, especially females, showed increased vulnerability to the adverse effects of pollution. Trends in lung capacity mirrored those in physical fitness, with outliers suggesting individual susceptibility. Notably, reduced green coverage was found to mediate 22% of the health impact of pollution (*p* < 0.05).

**Conclusion:**

This study highlights the disproportionate health impact of air pollution on college students, emphasizing the need for policies that focus on reducing emissions, expanding campus greenery, and promoting health education. Future research should incorporate individual fixed effects and broaden the study to include a wider range of regions and universities.

## 1 Background

Air pollution is one of the most serious environmental health risks globally. According to the World Health Organization, environmental air pollution is estimated to cause 4.2 million premature deaths worldwide in 2019 (1, 2). It is emphasized that particulate matter pollutants such as PM_2.5_ in the air pose significant health hazards. Particulate matter (PM) refers to suspended particles in the air with a diameter less than or equal to 2.5μm (3). These particles come from sources such as vehicle exhaust, industrial emissions, and the combustion of coal and fuel. Due to their small particle size and light weight (4), they can remain suspended in the air for a long time, altering the refractive index of light in the atmosphere and reducing air visibility. Furthermore, they typically enter the body through the lungs, transfer to various organs, and accumulate, causing inflammation and having a significant impact on health and the environment (5). Numerous studies have shown that prolonged exposure to pollutants can lead to airway inflammation and spasms, affecting respiratory function (6), Severe air quality pollution significantly increases the incidence and mortality of diseases such as respiratory, cardiovascular, and acute myocardial infarction, seriously harming human health and reducing life expectancy (7).

The impact of air pollution on human health is a problem of common concern across disciplines worldwide. From the perspective of natural sciences, the adverse effects of air pollutants on human health have been extensively studied (8, 9). Fields such as environmental science and pathology have unique advantages in this regard. However, natural sciences often struggle to address the economic and social factors behind environmental health issues and their impacts, and overlooking these factors may result in environmental health policies lacking specificity and effectiveness. In contrast, economics has the ability to translate endogenous mechanisms into real policy implications and provide relevant insights by integrating various social factors.

Grossman theoretically establishes the relationship between air pollution and health by incorporating the physiological law of individual health deterioration with age into the residents’ health production function (10) and Cropper further introduces air pollution into the model, thus establishing the link between the environment and health (11). One study (12) combine theoretical models with empirical data, verifying Grossman’s model conclusions and finding that air pollution accelerates individual health depreciation from physiological, psychological, and social adaptability perspectives, with long-term effects. Some scholars have constructed a model to measure the environmental and social health costs of economic growth and found that the substitution effect of economic growth on residents’ health is far greater than the income effect, leading to an overall decline in social health levels (13–15). Other scholars have further found from the perspective of social equity that due to heterogeneity of residents, health problems caused by air pollution have led to more health inequality (16, 17).

Although the above work has been completed, there are still gaps in the scientific literature. Previous studies have focused primarily on the impact of air pollution on physical activity in the United States, with limited research on middle-income countries like China. In terms of air quality monitoring, the United States began early and has accumulated extensive experience. Additionally, the United States has transitioned from being one of the regions with severe air pollution to one of the areas with the best air quality in the world (18). However, since the reform and opening up, rapid economic development in China has led to significant air pollution problems, posing a major threat to public health in the country. According to data from the World Health Organization, air pollution causes approximately 2 million deaths in China every year (19). Furthermore, the impact of air quality on the health of young people has not been explored. Existing literature has concentrated on the older adult population, finding a significant long-term negative impact of air pollution on the health of middle-aged and older individuals, with more pronounced effects on women, residents in northern regions, and those with lower education and income levels (20–22). The prevalence of diseases among middle-aged and older adult individuals is higher in areas with poorer air quality (23, 24). Additionally, air pollution can affect the mental health of middle-aged and older adult individuals by harming their physical health (20, 25). To address long-term factors, some studies have explored the immediate health effects of pollution on infant birth outcomes (26, 27). Previous research has investigated the longitudinal relationship between air pollution levels and changes in weekly physical activity among freshmen at Peking University in China. However, the health behaviors of students were self-reported, and the sample sizes were small, which may have been influenced by social desirability bias. Consequently, there are currently no follow-up large-sample studies on the impact of air quality on the physical health of university students. This study aims to construct a mixed cross-sectional econometric model to analyze the impact of atmospheric pollutants on the health of college students using physical fitness test data from a university in Xi’an. We hypothesize that air quality negatively affects the health and lung function of college students.

## 2 Data and methods

### 2.1 Sample selection and data source

“The National Student Physical Health Standards” is the fundamental guiding document and basic standard of education quality for China’s national school education work, which is an important basis for evaluating students’ comprehensive quality (28). University students need to participate in regular physical health tests every academic year. This test has been included as part of the talent training program, comprehensively evaluating students’ physical health level in terms of body shape, physical function, and physical fitness. Specific items include height, weight, lung capacity, 50-meter run, sit and reach, standing long jump, etc., and the scores are summarized according to the national prescribed indicators. The specific score calculation method is strictly calculated according to the individual indicators and weights of the national student physical health standards (29). Teachers will collect data in physical education classes. The attainment of students’ physical test results directly affects graduation (30). This study collected the physical test data of university students from this university from 2019 to 2022. Due to the fact that the participants come from different regions across the country, considering the impact of local air pollution on their physical test results, they should have resided in Xi’an for a certain period of time. Therefore, this study selected senior students as the research sample. There are a total of 21,580 research samples.

Xi’an is one of the typical large cities in the northwest region of China, facing multiple challenges of air pollution such as industrial emissions, vehicle exhaust emissions, and coal combustion. The air pollution is relatively serious with many universities. Therefore, Xi’an was chosen as the research sample. The air pollution data mainly comes from the Air Quality Index (AQI) published by the Chinese Ministry of Ecology and Environment, which is widely used to measure the degree of air pollution (31–33). The temperature and wind speed data come from the Chinese meteorological dataset, and the greening coverage data in the built-up areas come from the China City Statistical Yearbook and the Shaanxi Statistical Yearbook. Data processing was carried out using STATA 17.0 software, Excel software, etc. This study manually matched individual data according to cities with air pollution data and temperature and wind speed data.

### 2.2 Research methodology

#### 2.2.1 Kernel density estimation

This paper employs non-parametric kernel density estimation to analyze the dynamic evolution of college students’ physical fitness scores and vital capacity scores across different years. This research method effectively captures the changing trends and distributions of college students’ physical health, enabling a more comprehensive analysis of their current physical well-being.

This paper analyzes the dynamic evolution trend of college students’ physical fitness test scores and lung capacity scores in different years using non-parametric Kernel density estimation. Kernel density estimation has a weak dependence on the model and good statistical properties, with important applications in studying spatial distribution inhomogeneity. Suppose the density function of the random variable *X* is in the form of Equation 1:


(1) $$f\left(x\right)=\frac{1}{Nh}\sum_{i=k}^{N}k\left(\frac{x_{i}-x}{h}\right)$$

Equation 1 is the general formula for kernel density estimation, $f\left(x\right)$ represents the density function of the individual test scores and lung capacity scores, *N* is the number of observations, $X_{i}$ represents independently identically distributed observations, Kis the kernel function, his the bandwidth parameter. A larger h leads to a smoother density curve and larger estimation bias. Therefore, this study will choose a smaller bandwidth while ensuring a smooth curve.


(2) $$\left\{ \begin{array}{c} \lim k\left(x\right),x=0\\ \int_{-\infty }^{+\infty }k\left(x\right)dx=1,k\left(x\right)>0\\ \int_{-\infty }^{+\infty }k^{2}\left(x\right)dx<+\infty ,\sup k\left(x\right)<+\infty \end{array}\right.$$

Kernel function is a weighted function or a smoothing transformation function, which needs to satisfy the conditions as shown in Equation 2. Kernel functions include triangular kernel function, rectangular kernel function, Gaussian kernel function, etc. Empirical studies have shown that the fewer group data used, the greater the likelihood of selecting the Gaussian kernel function (34). Therefore, this study chooses the Gaussian kernel function to estimate the distribution dynamics and evolution trends of university students’ physical fitness test scores and lung capacity scores.

### 2.3 Variable description and research design

#### 2.3.1 Variable description

##### 2.3.1.1 Meaning of variables

Explanatory variables: based on existing research (35), atmospheric pollutants are measured using the Air Quality Index (AQI) published by the Ministry of Ecology and Environment of China. This index is primarily composed of the weighted average values of six major pollutants: PM_2.5_, PM_10_, SO_2_, CO, NO_2_, O_3_. The AQI ranges from 0 to 500, with higher values indicating more severe air pollution.

Explained variable: college students’ health is measured by the comprehensive score of college students’ physical fitness test (CS).

Control variables: in addition to the impact of air pollutants on the physical health of college students, individual factors and other environmental influences may also play a significant role. Therefore, this study draws on existing research to select gender, weight, height, and other factors as control variables for individual differences (36–37). Variations in gender, weight, and height can influence the Body Mass Index (BMI) related to health and may also affect individual perceptions of the external environment. Therefore, these factors are considered important control variables in our study. Additionally, average wind speed and average temperature were chosen as control variables for the external environment. Wind speed significantly impacts the natural environment, subsequently affecting human health. Higher temperatures can alter bodily functions and significantly influence human sensory perception, potentially posing harm to health (38).

Intermediary variable: based on the research of Satoshi and David (39), since atmospheric pollutants can significantly reduce the urban green area, the green area is selected as the intermediary variable and measured by the green coverage rate of the built-up area (GOA).

##### 2.3.1.2 Descriptive statistics

Table 1 presents the descriptive statistics of the main variables. The results indicate that the average value of College Students’ Physical Fitness (CS) is high; however, the data distribution exhibits a certain degree of variability, with a standard deviation of 9.62, suggesting significant individual differences. The minimum value is 10.2, while the maximum reaches 109, highlighting substantial individual variation in the physical health of college students.

**Table 1:** Descriptive statistics of variables.

Variable	Obs	mean	sd	min	max
CS	21,580	74.4815	9.6155	10.2	109
AQI	21,580	84.6045	4.3706	80.3611	92.5233
gender	21,580	0.3130	0.4637	0	1
Height	21,580	170.6616	8.3706	141.1	207
Weight	21,580	64.9426	13.4004	33.5	176.7
Temperatures	21,580	14.5233	0.3463	14.1464	15.0002
Windspeed	21,580	2.2416	0.2285	1.9666	2.4583
GOA	21,580	41.2233	1.447814	39.57	43.13

The average Air Quality Index (AQI) is 84.60, with a standard deviation of 4.37, indicating that the air quality index is relatively consistent within the sample, with a narrow range of fluctuation. The small difference between the minimum and maximum values suggests limited variation in air quality.

The gender variable is coded as 0 and 1, with a mean of 0.313 and a standard deviation of 0.464, indicating a relatively uniform gender distribution. The average height in the sample is 170.66 cm, with a standard deviation of 8.37 cm, demonstrating notable differences in height among individuals. The minimum height is 141.1 cm, while the maximum is 207 cm, reflecting a wide height range.

The average weight in the sample is 64.94 kg, with a standard deviation of 13.40 kg, indicating considerable variability in weight. The significant difference between the minimum and maximum values illustrates the extensive distribution of weights among participants.

The average temperature is 14.52°C, with a standard deviation of 0.35°C, indicating that temperatures in the sample are highly concentrated, with minimal fluctuations. Similarly, the average wind speed is 2.24 m/s, with a standard deviation of 0.23 m/s, suggesting that changes in wind speed are also minimal. The average value of Green Open Area (GOA) is 41.22, with a small standard deviation of 1.45, indicating that the green coverage rate is relatively stable and concentrated.

#### 2.3.2 Variance inflation factor test

To avoid the impact of multidisciplinary on the regression results, this study conducted VIF tests on the above variables. From Table 2, it can be seen that the VIF values of each variable are all less than 10, indicating no multidisciplinary issues.

**Table 2:** VIF test results.

Variable	VIF	1/VIF
Windspeed	7.35	0.136012
Temperatures	5.91	0.169203
Height	2.33	0.429783
Gender	1.94	0.515206
AQI	1.65	0.606926
Weight	1.52	0.655832
Mean VIF	3.45	

#### 2.3.3 Model construction

The study of the impact of environmental pollution on health originated from the health production function first established in 1972 to analyze the micro-health needs of residents (40). The function takes into account many factors including income method, health level, education, environment, etc. In addition to being affected by the environment, residents’ health is also affected by many other factors. On this basis, Cropper (11) and Gerking (41) continued to improve and perfect it. This study selected college students as a special group for individual research, aiming to analyze the impact of atmospheric pollutants on the health of college students, that is, whether the air quality index will affect the comprehensive scores of college students’ physical tests. Drawing on existing research, the mixed cross-sectional measurement model’s formula, as delineated in this paper, is represented by Equation 3 (38):


(3) $$CS_{\text{it}}=\alpha +\beta AQI_{\text{it}}+\gamma Z+\varepsilon_{\text{it}}$$

Among them, ${CS}_{\text{it}}$ represents the individual’s physical fitness test score for college students in 2017, ${AQI}_{\text{it}}$ represents the air quality index, Z represents the control variables, including the individual’s height, weight, gender, and the local average temperature and average wind speed, $\varepsilon_{\text{it}}$ which are random disturbance terms.

## 3 The dynamic evolution trend of college students’ physical fitness test scores and lung capacity scores

### 3.1 Physical fitness test results

**Figure 1:**
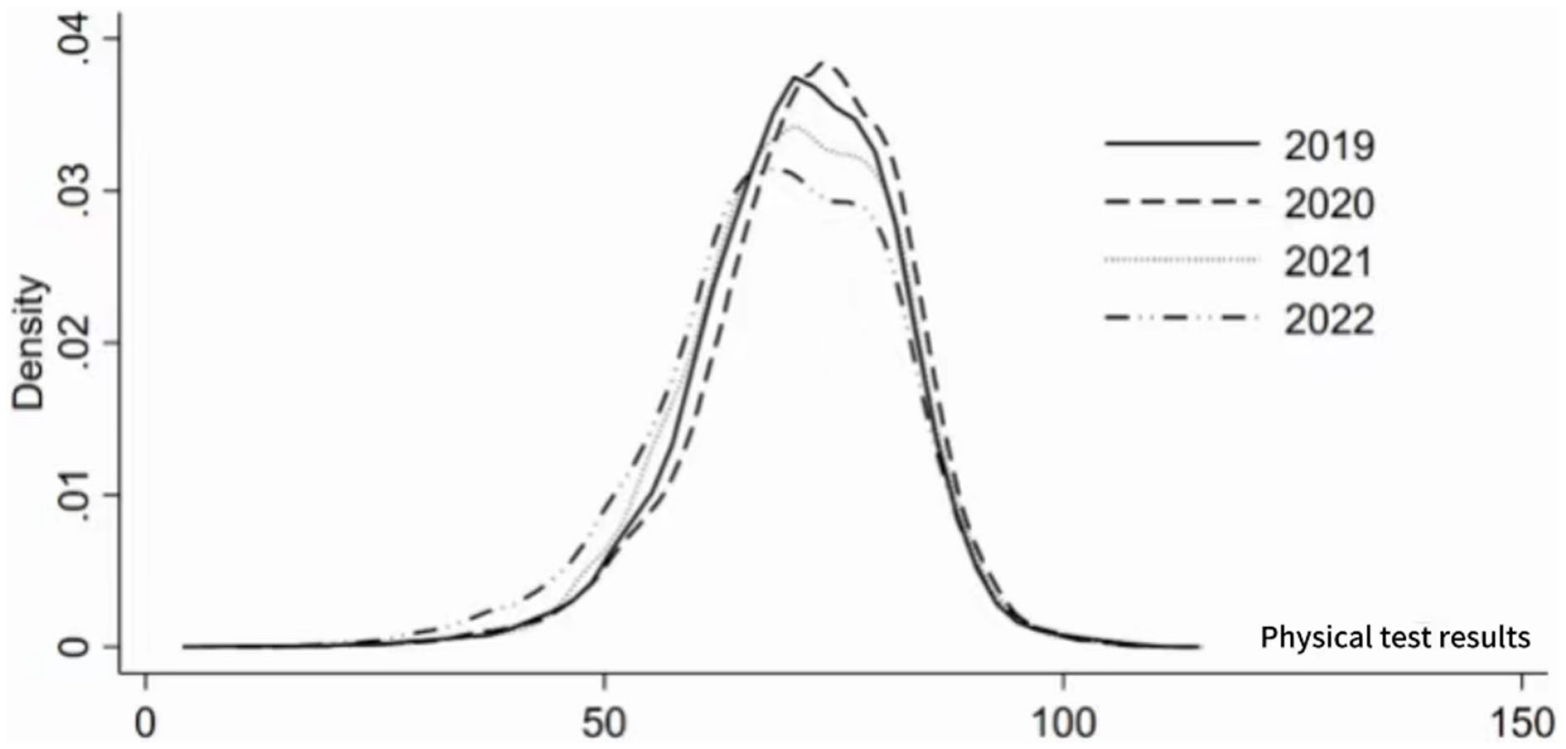
Kernel density estimation of physical fitness test scores for college students.

To analyze the dynamic evolution of the physical fitness test scores of college students in a university in Xi’an, this paper uses Kernel density estimation to analyze the distribution location, spread, and polarization trend within the sample period. The estimation results are shown in Figure 1. The kernel density curve shows: (1) From the perspective of distribution location, the center position of the curve first moves to the right and then to the left, indicating that the physical fitness test scores of the college students first rise and then fall. (2) From the perspective of distribution shape, the kernel density curve generally shows a decrease in the height of the main peak, and the overall width of the wave increases, indicating that the absolute difference in physical fitness test scores among the college students in the sample period is increasing. (3) From the perspective of distribution spread, there is no obvious right-skewness in the kernel density curve of the physical fitness test scores of the college students, indicating that there is no phenomenon where the physical fitness test score of one college student is much higher than that of another student among the subjects. (4) From the perspective of polarization, there is no obvious bimodal distribution in the kernel density curve of the physical fitness test scores of the college students, indicating that there is no obvious polarization phenomenon in the physical fitness test scores of the college students.

**Figure 2:**
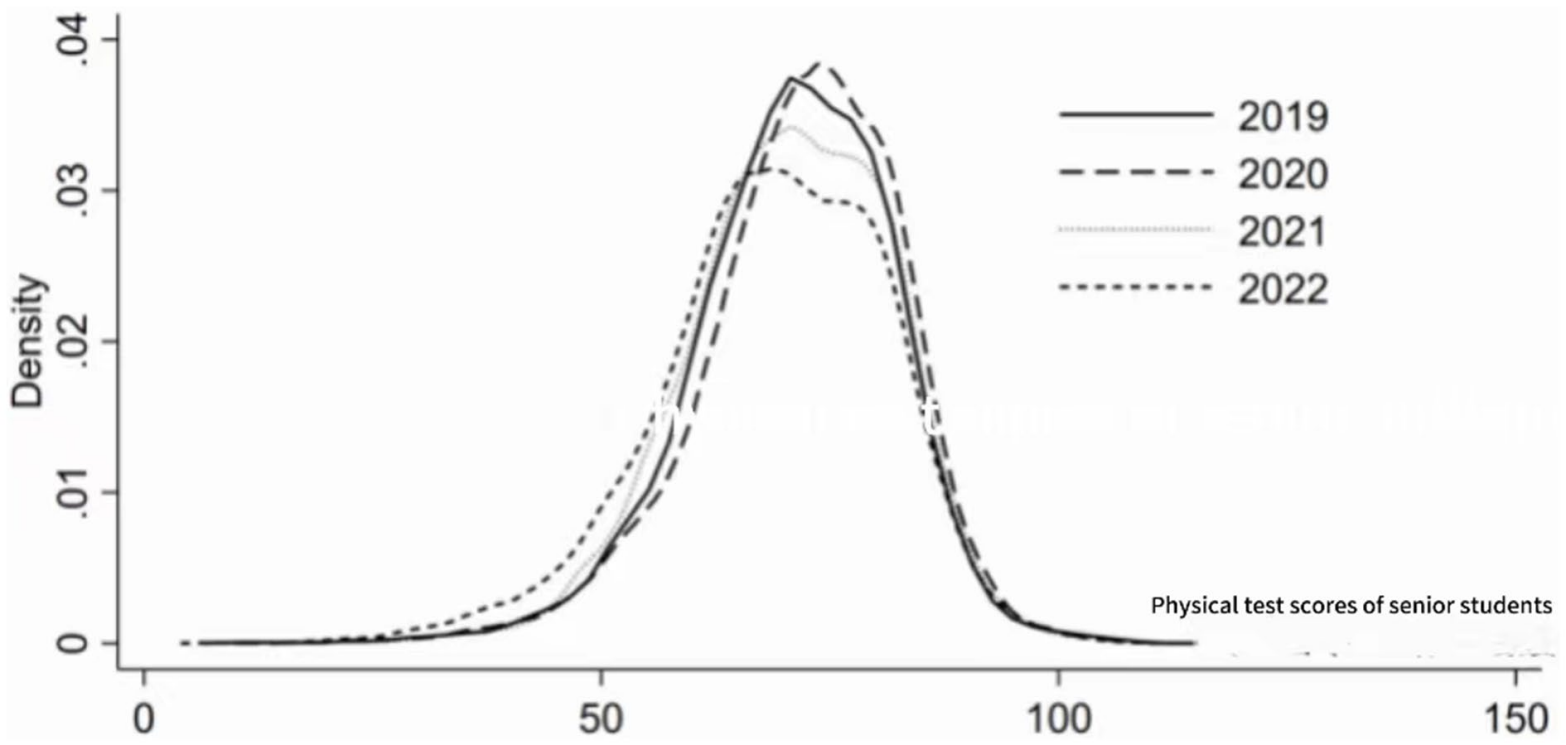
Density estimation of physical fitness test scores for senior students in the fourth year.

Further analysis of the dynamic evolution trends of the physical fitness test scores of fourth-year students estimates the results as shown in Figure 2. The kernel density curve shows: (1) From the perspective of distribution position, the center of the curve first moves to the right and then to the left, indicating that the physical fitness test scores of fourth-year students initially rise and then fall. (2) From the perspective of distribution shape, the overall performance of the kernel density curve shows a decrease in the height of the main peak and a slight increase in the width of the bandwidth, indicating a slight increase in the absolute difference in test scores of fourth-year students within the sample period. (3) In terms of distribution stretchiness, the kernel density curve of the physical fitness test scores of fourth-year students does not show a clear right tail phenomenon, indicating that there is no phenomenon in which the test scores of one college student are significantly higher than those of another student within the sample. (4) In terms of polarization, the kernel density curve of the physical fitness test scores of the fourth-year students does not exhibit a clear bimodal distribution, indicating that there is no clear polarization phenomenon in the physical fitness test scores of fourth-year students in the sample.

### 3.2 Lung capacity results

**Figure 3:**
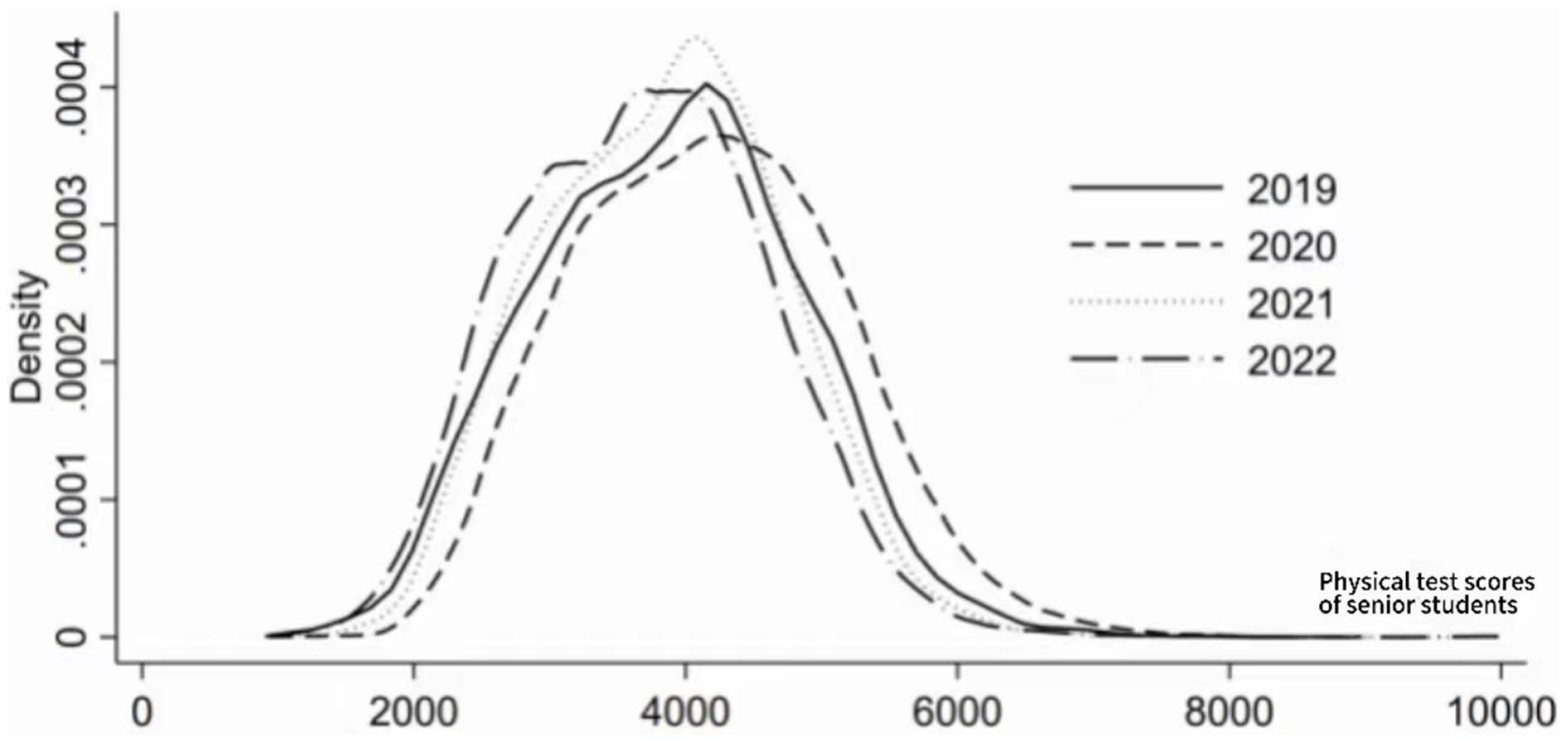
Density estimation of college students’ lung capacity scores.

For the analysis of the dynamic evolution of pulmonary function scores of college students at a university in Xi’an, this article uses Kernel density estimation to analyze the distribution position, spread, and polarization trend within the sample period. The estimated results are shown in Figure 3. The Kernel density curve shows: (1) from the perspective of distribution position, the center position of the curve first moves to the right and then to the left, indicating that the pulmonary function scores of the participating college students first increase and then decrease. (2) From the perspective of distribution shape, the Kernel density curve shows a slight increase in the overall height of the main peak, with no significant change in the width of the fluctuation, indicating that there is no significant change in the absolute difference in pulmonary function scores of the participating college students within the sample period. (3) In terms of distribution spread, the Kernel density curve of the pulmonary function scores of the participating college students shows a more obvious right tail phenomenon, indicating that there is a phenomenon where the pulmonary function scores of one student in the sample are much higher than those of another student. (4) In terms of polarization, the Kernel density curve of the pulmonary function scores of the participating college students does not show a clear bimodal distribution, indicating that there is no clear polarization phenomenon in the pulmonary function scores of the participating college students.

**Figure 4:**
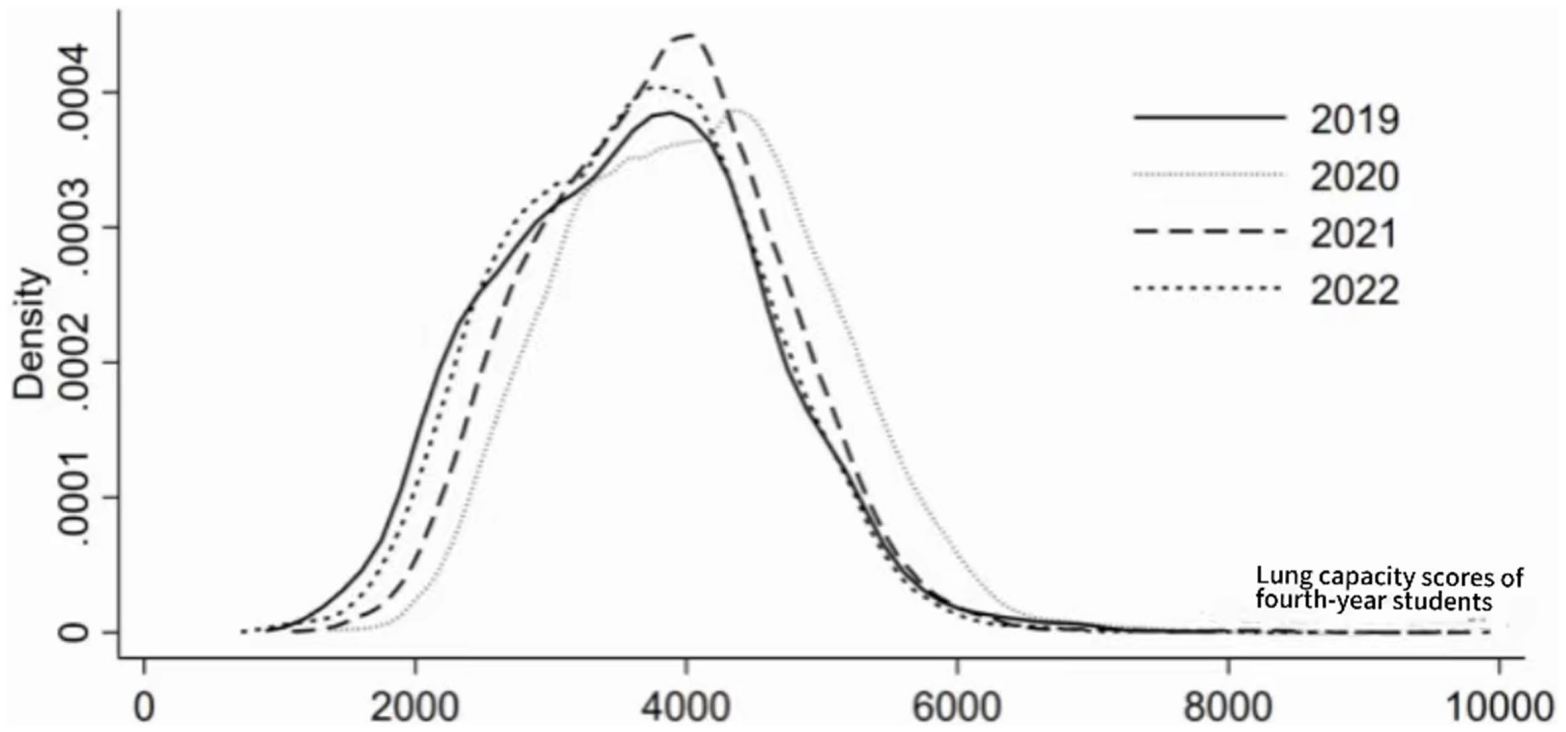
Density estimate of lung capacity scores for senior students.

Further analysis of the dynamic evolution trend of lung capacity scores of fourth-year students estimated the results as shown in Figure 4. The kernel density curve shows: (1) From the perspective of distribution position, the center position of the curve first moves to the right and then to the left, indicating that the lung capacity scores of fourth-year students initially rise and then fall. (2) From the perspective of distribution shape, the overall performance of the kernel density curve shows a slight increase, with no significant change in the width of the wave, indicating that there is no significant change in the absolute difference in lung capacity scores of fourth-year students within the sample period. (3) From the perspective of distribution flexibility, the kernel density curve of lung capacity scores of fourth-year students shows a more obvious right tail phenomenon, indicating that there are individuals among the participants whose lung capacity scores are significantly higher than those of other students. (4) From the perspective of polarization phenomenon, the kernel density curve of physical fitness scores of participants does not show a clear bimodal distribution, indicating that there is no clear polarization phenomenon in lung capacity scores of fourth-year students.

## 4 Empirical results analysis

### 4.1 Benchmark regression

Table 3 reports the empirical estimates of the impact of atmospheric pollutants on the health of college students. The AQI index is significantly negatively correlated with the comprehensive physical fitness scores of college students, indicating that atmospheric pollutants do have a significant impact on the health of college students. Specifically, controlling for individual’s height, weight, gender, as well as local average temperature and average wind speed, for every 1-unit change in the Air Quality Index (AQI), the physical fitness scores of college students will decrease by 0.1094 units. Among the control variables, the individual’s height, weight, and gender are significant at the 1% level, indicating that the control variables will also affect the differences in the subjects. The local average wind speed and average temperature have significant effects on the physical health of college students.

**Table 3:** Benchmark regression results.

Variables	CS
AQI	−0.1094***
	(−6.9113)
Windspeed	1.9222***
	(2.9771)
Temperatures	−2.4460***
	(−6.1464)
Gender	7.2205***
	(43.1107)
Height	0.3930***
	(35.5821)
Weight	−0.3385***
	(−57.0045)
Constant	67.6473***
	(14.2463)
Observations	21,545
R-squared	0.2694

***, **, *Represent significance levels of 1, 5, and 10%, respectively.

### 4.2 Robustness test

The AQI is composed of 5 pollutants, among which PM_2.5_, PM_10_, and CO have attracted attention from various sectors of society (42). Therefore, we use the 24-h moving average data of PM_2.5_, PM_10_, and CO at the prefecture level from the China National Environmental Monitoring Station to replace AQI for robustness testing. By replacing the core explanatory variables, the results are shown in Table 4. The results show that for every 1 unit change in PM_2.5__24h, the physical fitness test score of college students will decrease by 0.2643 units, which is still significant; for every 1 unit change in PM_10__24h, the physical fitness test score of college students will decrease by 0.0897 units, which is still significant. For every 1 unit change in CO_24h, the physical fitness test score of college students will decrease by 11.5438 units, which is still significant, but differs significantly from the benchmark regression results.

**Table 4:** Robustness test results.

Variables	CS	CS	CS
CO_24h			−11.5438***
			(−6.9113)
PM_10__24h		−0.0897***	
		(−6.9113)	
PM_2.5__24h	−0.2643***		
	(−6.9113)		
Windspeed	−288.4464***	5.3581***	2.9430***
	(−6.8103)	(9.3438)	(5.0540)
Temperatures	281.0532***	−4.4773***	−4.0825***
	(6.8311)	(−10.7563)	(−10.3086)
Gender	7.2205***	7.2205***	7.2205***
	(43.1107)	(43.1107)	(43.1107)
Height	0.3930***	0.3930***	0.3930***
	(35.5821)	(35.5821)	(35.5821)
Weight	−0.3385***	−0.3385***	−0.3385***
	(−57.0045)	(−57.0045)	(−57.0045)
Constant	−2,966.8917***	88.6312***	88.6332***
	(−6.7633)	(15.1243)	(15.1242)
Observations	21,545	21,545	21,545
R-squared	0.2694	0.2694	0.2694

***, **, *Represent significance levels of 1, 5, and 10%, respectively.

### 4.3 Heterogeneity analysis

Studies have shown that there are significant differences between males and females in areas such as physical fitness and specialization. Yang Jidong and Zhang Yiran (43) also found that air pollution has a greater negative impact on the happiness of males, while the impact on females is not significant. Further analysis is conducted on the heterogeneity of groups in terms of differences in health status when facing pollution impact. We conduct group regression analysis based on gender differences. Table 5 reports the response of male and female senior students to air pollutants. The results show that air pollutants have a significant negative impact on senior students of different genders, with each unit change in AQI leading to a change of 0.0708 units in physical fitness scores for male senior students, which is 0.116 units less than the change for female senior students, indicating that air pollutants have a greater impact on the comprehensive physical fitness scores of senior females.

**Table 5:** Heterogeneity analysis test results.

	Male	Female
Variables	CS	CS
AQI	−0.0708***	−0.1868***
	(−3.5202)	(−7.5460)
Windspeed	2.4933***	0.5891
	(3.0251)	(0.5978)
Temperatures	−2.7001***	−1.7924***
	(−5.3406)	(−2.9150)
Height	0.3929***	0.3826***
	(28.7413)	(21.3831)
Weight	−0.3521***	−0.2734***
	(−52.1744)	(−21.9000)
Constant	67.7523***	72.9639***
	(11.2176)	(10.1434)
Observations	14,799	6,746
R-squared	0.1997	0.1212

***, **, *indicate the significance levels of 5, 1, and 10%, respectively.

### 4.4 Analysis of influencing mechanisms

The analysis above confirms that atmospheric pollutants have a significant negative impact on the health of college students. By what mechanism do atmospheric pollutants affect the physical health of college students? Based on a comprehensive review of existing literature, this paper believes that atmospheric pollutants will affect the physical health of college students by influencing the green space area in urban areas.

To test the impact of atmospheric pollutants on the health of college students, this article takes atmospheric pollution as the core explanatory variable, replaces the dependent variable of model (1) with the green coverage rate of built-up areas, and examines the impact of atmospheric pollutants on the mechanism variables. Table 6 verifies the green coverage rate of built-up areas as a pathway for atmospheric pollutants to affect the health of college students. The results indicate that an increase in atmospheric pollutants will reduce the green coverage area of built-up areas. Severe atmospheric pollution will decrease the green coverage area of urban areas, which will in turn affect human health. The analysis above partially confirms the mechanism by which atmospheric pollutants affect the health of college students.

**Table 6:** Mechanism examination of air pollutants on the physical health of college students.

Variables	GCA
AQI	−0.2296***
	(−9.2727e+13)
Windspeed	−14.1229***
	(−1.4463e+14)
Temperatures	9.5457***
	(2.2177e+14)
Gender	−0.0000***
	(−3.3379)
Height	−0.0000***
	(−8.2092)
Weight	0.0000***
	(4.3344)
Constant	−46.3287***
	(−9.5838e+13)
Observations	21,545
R-squared	1.0000

***, **, *indicate the significance levels of 5, 1, and 10%, respectively.

## 5 Discussion

This study aims to investigate the impact of atmospheric pollutants on college students’ physical health and lung function, with a particular focus on how air pollution affects their physical fitness test scores and lung capacity. To achieve this, we employed kernel density estimation to systematically analyze the trends in physical fitness test scores among college students from 2019 to 2022. The study revealed fluctuations in the health status of students, especially significant changes in lung function, suggesting that air pollution has a profound effect on their physical well-being.

First, we observed a “first increase, then decrease” fluctuation pattern in students’ physical fitness test scores. The kernel density estimation results indicate that while overall test scores exhibited some volatility, the disparities between scores gradually expanded, with no signs of extreme polarization in individual health outcomes. In other words, although some students showed significant improvements in fitness, the overall trend remained stable, without a clear polarization effect. This fluctuation may be linked to the seasonal variation in air pollution levels. For instance, spring and autumn are typically periods of more severe air pollution, which could have a temporary adverse impact on students’ physical fitness and health. However, whether long-term exposure to air pollution leads to lasting health deterioration requires further longitudinal research for confirmation. Overall, these findings suggest that the relationship between air pollution and college students’ health is not a simple linear one, but rather follows a complex, fluctuating pattern.

Further analysis revealed that lung capacity scores of upper-year students also followed a “first increase, then decrease” trend. Similar to the volatility observed in overall fitness scores, lung capacity scores exhibited significant fluctuations during certain periods. We hypothesize that as students advance in their academic years, their academic pressure and lifestyle changes may partially mitigate the direct effects of air pollution on lung function. However, during periods of high air pollution, particularly in the autumn and winter, upper-year students’ lung capacity might experience more direct negative effects. This finding prompts consideration of the potential mechanisms underlying the relationship between air pollution and lung function. Air pollution, particularly fine particulate matter (PM_2.5_) and nitrogen dioxide (NO_2_), has been shown to have negative impacts on respiratory health. Upper-year students, who may be exposed to more air pollution due to high-intensity academic work and campus activities, could experience accelerated declines in lung function. Therefore, despite some improvements in lung capacity among certain upper-year students, the impact of air pollution remains significant, especially in environments with high pollution concentrations.

Baseline regression analysis further revealed the relationship between individual characteristics (such as height, weight, and gender) and health outcomes. Physiological features like height and weight clearly influence physical fitness test results, while gender appears to moderate the effect of air pollution on health. Notably, female students, due to physiological and metabolic differences, may be more susceptible to the negative effects of air pollution. The study found that in areas with higher pollution levels, the decline in fitness among female students was significantly more pronounced compared to their male counterparts. This gender disparity warrants further attention.

In addition to individual characteristics, local meteorological factors such as wind speed and temperature also played a role in influencing health outcomes. High temperatures and low wind speeds tend to exacerbate the stagnation of air pollutants, leading to elevated concentrations of pollution. The interaction of these environmental factors, particularly in relation to seasonal variations in air pollution, further underscores the complexity of the impact of air pollution on college students’ health.

To ensure the robustness of our findings, we conducted additional sensitivity tests and heterogeneity analyses. Although the sensitivity tests consistently showed a significant impact of air pollution on students’ physical health, we observed noteworthy differences across various groups. Particularly, the effect of air pollution on female students’ physical fitness test scores was more significant. This may be due to physiological differences, variations in daily activity patterns, and differing levels of exposure to polluted environments, all of which make female students more vulnerable to the adverse effects of air pollution. The importance of this heterogeneity analysis lies in its potential to inform future health interventions. Targeted health protection measures for high-risk groups, such as upper-year female students, may be necessary. These could include reducing outdoor activities, improving indoor air quality, and increasing air pollution monitoring, especially during periods of higher pollution levels, to provide timely health alerts for these vulnerable populations.

This study shows that the lung capacity scores of senior female university students exhibit a trend of first increasing and then decreasing with changes in air pollution levels, with no significant polarization in score distribution, indicating that the individual differences are relatively small. This finding aligns with existing literature, where many studies indicate that the impact of air pollution on lung function is gradual and cyclical, and long-term exposure to pollution may lead to a decline in lung function (44, 45). The fluctuations in lung capacity scores in this study reflect the temporal dynamics of the health impacts of air pollution (46–48), suggesting that changes in pollution levels at different times have varying degrees of impact on students’ lung function. Additionally, during specific periods, the distribution of scores broadened, and the absolute gaps increased. Although there was no evident polarization, the widening disparity still suggests a significant impact of air pollution on students’ health. Relevant research has pointed out that long-term exposure to air pollution may lead individuals to reduce outdoor activities, which in turn results in a decline in physical fitness and accelerates the deterioration of cardiovascular and respiratory health (49, 50). Air pollution not only has a direct impact on physical fitness but also indirectly affects health by altering lifestyle behaviors, such as reduced physical activity (51), increased sedentary time, and changes in dietary patterns (52, 53). The fluctuations in physical fitness scores and the widening distribution gaps observed in this study may reflect these indirect effects.

Furthermore, the impact of air pollution on health is not limited to physical fitness but also involves other aspects such as mental health, sleep quality, and dietary habits (54, 55). Studies have shown that individuals living in high-pollution environments tend to choose unhealthy, high-calorie foods, further impacting overall health (56–58). Moreover, air pollution may affect sleep quality and increase the risk of anxiety and depression (59, 60). Although this study did not specifically explore mental health and sleep quality, the observed fluctuations in physical fitness scores and overall health suggest that air pollution has a profound impact on students’ physical and mental well-being, especially with prolonged exposure (61).

In summary, the results of this study are consistent with existing literature, indicating that air pollution not only directly impacts students’ physical health but also indirectly affects their physical fitness and mental health through changes in lifestyle (62) and social behaviors (63). These findings offer valuable insights into the multifaceted impact of environmental pollution on the health of university students and provide a scientific basis for future policy-making aimed at improving air quality and enhancing students’ health.

The study’s strength lies in its use of fitness assessment data for cross-sectional research, leveraging large sample sizes and multidimensional data to enhance the representatives and reliability of the findings. Moreover, since the assessment measures are generally straightforward and objective, they minimize the influence of subjective factors. However, the study also has notable limitations. First, the sample’s representatives is limited as it only includes students from one university, which may not fully represent the broader undergraduate population in China or other developing countries. Thus, there is a need for broader replication across various Chinese universities. Second, regional differences in air quality may affect the results. The air pollution data, based on the AQI published by the Ministry of Ecology and Environment of China, may not account for significant local variations within the expansive city of Xi’an, Shaanxi. Future research should incorporate individual fixed-effects models to control for time-invariant factors that may confound the results. Additionally, multivariate regression analysis of highly correlated dependent variables may introduce potential errors. Previous research indicates that even without exposure to air pollution, freshmen experience a decline in physical activity over their first year (64). There is also a need for better control of other potential influencing factors, such as diet (65), lifestyle, and genetics, which may impact student health but were not adequately considered in this study.

## 6 Conclusion

This study explored the impact of air pollution on the physical health and lung function of college students. By using the Kernel density estimation method, the dynamic changes in the physical fitness and lung capacity scores of college students at a certain university were analyzed. The results showed that during the sample period, the physical fitness scores of the college students first rose and then fell, with the absolute difference gradually increasing, but there was no phenomenon of one student far surpassing others, nor was there an obvious polarization phenomenon. At the same time, the lung capacity scores of the college students also showed a trend of first rising and then falling, with no significant change in the absolute difference, but there were individual students whose lung capacity scores far exceeded others. Further analysis of the changes in physical fitness scores and lung capacity scores of fourth-year students showed that, during the sample period, the physical fitness scores of fourth-year students first rose and then fell, with a slight increase in the absolute difference, and there was no phenomenon of one student far surpassing others, nor was there an obvious polarization phenomenon. Similarly, the lung capacity scores of fourth-year students also showed a trend of first rising and then falling, with no significant change in the absolute difference, but there were individual students whose lung capacity scores far exceeded others. By using mixed cross-sectional data and econometric models, the impact of air pollution on the health of college students was analyzed. The results showed that the negative impact of air pollution on the health of fourth-year students was significant, even after multiple robustness tests. Further heterogeneity tests on gender differences showed that the impact of air pollution on the health of fourth-year female students was greater than that on male students. Mechanism tests showed that air pollution affects the physical health of college students by influencing the green coverage rate in built-up areas. This indicates that air pollution has a certain harmful effect on the health of fourth-year college students.

Based on the research results of this study, aiming to promote environmental health work and strengthen disease management to improve the physical health and lung function of university students, the following policy suggestions are proposed: (1) Improve air quality: The government should strengthen air quality monitoring and management in the surrounding areas of university campuses, take measures to reduce the emissions of atmospheric pollutants, including enhancing purification measures for factory and traffic exhaust, and formulating stricter environmental regulations, etc. (2) Increase green coverage: The government and schools should increase the construction and maintenance of greenery on university campuses, increase green coverage. This can be achieved by planting more trees and lawns, establishing gardens and parks, etc. to enhance green coverage. (3) Strengthen health education: Schools should strengthen health education for university students, increase students’ awareness and understanding of the impact of air pollution on health. Health lectures, promotional activities, etc. can be conducted to enhance students’ awareness of their health and protection. (4) Promote sports: Schools should encourage and promote sports, provide more sports facilities and venues, and organize various sports activities. Sports can help improve the physical fitness and lung function of university students, alleviate the negative impact of air pollution on the body.

Future research should replicate the study in other cities and universities in China to further validate our findings. Meanwhile, urgent policy interventions are needed to reduce the level of air pollution in China. To eliminate the confounding bias of time-invariant factors within participants, future studies plan to introduce individual fixed effects models. Additionally, in terms of controlling variables, subsequent research will focus on addressing multidisciplinary issues and further explore the more definitive relationship between air pollution and physical activity behaviors among college freshmen.

## Data Availability

The original contributions presented in the study are included in the article/supplementary material, further inquiries can be directed to the corresponding authors.
