# Correction: The impact of atmospheric pollutants on the physical health of college students based on physical examination data of college students from a university in Xi'an, Shaanxi Province, China

**DOI:** 10.3389/fpubh.2025.1626981

**Published:** 2025-08-11

**Authors:** Jiaxin He, Ke Liu, Zhiyu He

**Affiliations:** ^1^International Business School, Shaanxi Normal University, Xi'an, Shaanxi, China; ^2^Physical Education and Sports Science Center, Xi'an Jiaotong University, Xi'an, Shaanxi, China

**Keywords:** air quality, physical health of college students, health effects, physical measurement data, pulmonary function of college students

In the published article, there was an error in the author list, and author Jiaxin He was erroneously included as a Corresponding Author. The corrected author list appears below.

Jiaxin He^1†^, Ke Liu^1†^ and Zhiyu He^2^*^^

In the published article, there was an error in the article title. Instead of “The impact of atmospheric pollutants on the physical health of college students—based on physical examination data of college students from a certain university in Xi'an, Shaanxi Province, China”, it should be “The impact of atmospheric pollutants on the physical health of college students based on physical examination data of college students from a university in Xi'an, Shaanxi Province, China”.

In the published article, these references were erroneously included and should be removed:

37. You, Y. Accelerometer-measured physical activity and sedentary behaviour are associated with C-reactive protein in US adults who get insufficient sleep: a threshold and isotemporal substitution effect analysis. *J Sports Sci*. (2024) 42:527–36. doi: 10.1080/02640414.2024.2348906

38. You, Y, Li, J, Zhang, Y, Li, X, Li, X, and Ma, X. Exploring the potential relationship between short sleep risks and cognitive function from the perspective of inflammatory biomarkers and cellular pathways: insights from population-based and mice studies. *CNS Neurosci Ther*. (2024) 30:e14783. doi: 10.1111/cns.14783

43. You, Y, Liu, J, Li, X, Wang, P, Liu, R, and Ma, X. Relationship between accelerometer-measured sleep duration and Stroop performance: a functional near-infrared spectroscopy study among young adults. *PeerJ*. (2024) 12:e17057. doi: 10.7717/peerj.17057

54. You, Y, Ablitip, A, Lin, Y, Tang, M, Qian, W, Zhang, D, et al. Threshold effect of physical exercise on its association to diabetes mellitus in short sleep population: evidence from a nationwide study. *Front Endocrinol*. (2024) 15:1437452. doi: 10.3389/fendo.2024.1437452

55. You, Y, Ablitip, A, Chen, Y, Ding, H, Chen, K, Cui, Y, et al. Saturation effects of the relationship between physical exercise and systemic immune inflammation index in the short-sleep population: a cross-sectional study. *BMC Public Health*. (2024) 24:1920. doi: 10.1186/s12889-024-19432-7

56. You, Y, Chen, Y, Wei, M, Tang, M, Lu, Y, Zhang, Q, et al. Mediation role of recreational physical activity in the relationship between the dietary intake of live microbes and the systemic immune-inflammation index: a real-world cross-sectional study. *Nutrients*. (2024) 16:777. doi: 10.3390/nu16060777

57. You, Y, Wang, R, Li, J, Cao, F, Zhang, Y, and Ma, X. The role of dietary intake of live microbes in the association between leisure-time physical activity and depressive symptoms: a population-based study. *Appl Physiol Nutr Metab*. (2024) 49:1014–24. doi: 10.1139/apnm-2023-0550

59. You, Y, Mo, L, Tong, J, Chen, X, and You, Y. The role of education attainment on 24-hour movement behavior in emerging adults: evidence from a population-based study. *Front Public Health*. (2024) 12:1197150. doi: 10.3389/fpubh.2024.1197150

In the published article the following reference “Clougherty JE. A growing role for gender analysis in air pollution epidemiology. *Environ Health Perspect*. (2010) 118:167–176. doi: 10.1289/ehp.0900994;

Chen L, Gao D, Ma T, Chen M, Li Y, Ma Y, et al. Could greenness modify the effects of physical activity and air pollutants on overweight and obesity among children and adolescents? *Sci Total Environ*. (2022) 832:155117. doi: 10.1016/j.scitotenv.2022.155117” was not cited in the article. The citation has now been inserted in **[2 Data and methods]**, [*2.3.1.1 Meaning of variables*], [paragraph 3] and should read:

“Control variables: in addition to the impact of air pollutants on the physical health of college students, individual factors and other environmental influences may also play a significant role. Therefore, this study draws on existing research to select gender, weight, height, and other factors as control variables for individual differences (1, 2).”

In the published article

“Bernard P, Chevance G, Kingsbury C, Baillot A, Romain AJ, Molinier V, et al. Climate change, physical activity and sport: a systematic review. *Sports Med*. (2021) 51:1041–59. doi: 10.1007/s40279-021-01439-4;

Yu H, Zhang H. Impact of ambient air pollution on physical activity and sedentary behavior in children. *BMC Public Health*. (2023) 23:357. doi: 10.1186/s12889-023-15269-8” was not cited in the article. The citation has now been inserted in **[Discussion]**, [paragraph 9] and should read:

“Air pollution not only has a direct impact on physical fitness but also indirectly affects health by altering lifestyle behaviors, such as reduced physical activity (53), increased sedentary time, and changes in dietary patterns (3, 4). The fluctuations in physical fitness scores and the widening distribution gaps observed in this study may reflect these indirect effects.”

In the published article

“Wang L, Zhang J, Wei J, Zong J, Lu C, Du Y, et al. Association of ambient air pollution exposure and its variability with subjective sleep quality in China: a multilevel modeling analysis. *Environ Pollut*. (2022) 312:120020. doi: 10.1016/j.envpol.2022.120020;

Liu J, Zou P, Ma Y. The effect of air pollution on food preferences. *J Acad Mark Sci*. (2022) 50:410–23. doi: 10.1007/s11747-021-00809-8” was not cited in the article. The citation has now been inserted in **[Discussion]**, [paragraph 9] and should read:

“Furthermore, the impact of air pollution on health is not limited to physical fitness but also involves other aspects such as mental health, sleep quality, and dietary habits (5, 6). Studies have shown that individuals living in high-pollution environments tend to choose unhealthy, high-calorie foods, further impacting overall health (58) (7)]”

The original version of this article has been updated.

## References

[1] Clougherty JE. A growing role for gender analysis in air pollution epidemiology. Environ Health Perspect. (2010) 118:167–176. 10.1289/ehp.0900994 20123621 PMC2831913

[2] Chen L Gao D Ma T Chen M Li Y Ma Y. Could greenness modify the effects of physical activity and air pollutants on overweight and obesity among children and adolescents? Sci Total Environ. (2022) 832:155117. 10.1016/j.scitotenv.2022.155117 35398425

[3] Bernard P Chevance G Kingsbury C Baillot A Romain AJ Molinier V. Climate change, physical activity and sport: a systematic review. Sports Med. (2021) 51:1041–59. 10.1007/s40279-021-01439-4 33689139

[4] Yu H Zhang H. Impact of ambient air pollution on physical activity and sedentary behavior in children. BMC Public Health. (2023) 23:357. 10.1186/s12889-023-15269-8 36803326 PMC9936470

[5] Wang L Zhang J Wei J Zong J Lu C Du Y. Association of ambient air pollution exposure and its variability with subjective sleep quality in China: a multilevel modeling analysis. Environ Pollut. (2022) 312:120020. 10.1016/j.envpol.2022.120020 36028077

[6] Liu J Zou P Ma Y. The effect of air pollution on food preferences. J Acad Mark Sci. (2022) 50:410–23. 10.1007/s11747-021-00809-8

[7] Chen Z Herting MM Chatzi L Belcher BR Alderete TL McConnell R. Regional and traffic-related air pollutants are associated with higher consumption of fast food and trans fat among adolescents. Am J Clin Nutr. (2019) 109:99–108. 10.1093/ajcn/nqy232 30596809 PMC6358030

